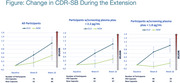# Impact of AD Co‐Pathology on Response to Neflamapimod (p38α Kinase Inhibitor) in Patients with Dementia with Lewy Bodies

**DOI:** 10.1002/alz70861_108885

**Published:** 2025-12-23

**Authors:** John J Alam, Nathalie Adda, Charlotte E. Teunissen

**Affiliations:** ^1^ CervoMed Inc, Boston, MA USA; ^2^ CervoMed, Boston, MA USA; ^3^ Neurochemistry Laboratory, Department of Laboratory Medicine, Amsterdam Neuroscience, Amsterdam UMC, Vrije Universiteit Amsterdam, Amsterdam Netherlands

## Abstract

**Background:**

Neflamapimod (oral drug that targets cholinergic dysfunction and degeneration) was recently reported in the RewinD‐LB Phase 2b study in patients with DLB without AD co‐pathology to meaningfully impact clinical progression, assessed by CDR‐SB. Exclusion criteria for AD co‐pathology (≥ 2.4 pg/mL plasma ptau181 at screening) in RewinD‐LB was based on evaluation of data from an AD dementia cohort. Herein, we evaluate the impact of different plasma ptau181 cut‐off levels to define AD co‐pathology.

**Method:**

16‐week randomized (1:1), placebo‐controlled study (“Initial Phase”), with 32‐week neflamapimod‐only Extension. The study was not able to effectively evaluate neflamapimod treatment effects in the Initial Phase because dosing with the capsules utilized in that phase (“Old Capsules”) did not lead to targeted plasma drug concentrations. With introduction in the Extension of a new batch of capsules that achieved the targeted plasma drug concentrations, the study was able to evaluate the effects of treatment with New Capsules (*N* =94; active drug arm) during the 1st 16 weeks of the Extension against outcomes in participants who continued to receive Old Capsules (*N* =55), which served as a control arm. In the current analysis, treatment effects were evaluated in: (1) All participants; (2) Subset with screening plasma ptau181<2.2 pg/mL, the cut‐off utilized to analyze phase 2a; (3) Subset with screening plasma ptau181<1.8 pg/mL (Youden’s cut‐off for CSF ptau181 or CSF tau in the AMC DLB cohort).

**Result:**

For all three subsets, there is less clinical worsening (assessed by change in CDR‐SB with New Capsules compared to Old Capsules, with progressively less worsening with New Capsules across the three subsets (Figure). Moreover, in a within‐subject comparison of change in CDR‐SB over 16 weeks in participants who received placebo during the Initial phase and ≥ 12 weeks New Capsules in the Extension, there was significant improvement with New Capsules in <2.2 pg/mL subset (+0.77 w/placebo vs. +0.21 w/New Capsules, *p* =0.044) and in <1.8 pg/mL subset (+0.95 w/placebo vs. ‐0.17 w/New Capsules, *p* =0.005).

**Conclusion:**

The results further demonstrate that neflamapimod beneficially impacts clinical progression in patients with DLB and confirm with larger participant numbers the phase 2a finding that AD co‐pathology impacts response to neflamapimod treatment.